# Frequency of intra-aortic balloon pump insertion and associated factors in coronary artery bypass Grafting in a tertiary care hospital

**DOI:** 10.12669/pjms.37.2.3614

**Published:** 2021

**Authors:** Imtiaz Ahmad, Mujahid Ul Islam, Mujeeb Ur Rehman, Bahauddin Khan

**Affiliations:** 1 Dr. Imtiaz Ahmad, FCPS. Associate Professor, Department of Anesthesia, Northwest General Hospital and Research Center, Peshawar, Pakistan; 2 Dr. Mujahid Ul Islam, FCPS. Associate Professor, Department of Anesthesia, Northwest General Hospital and Research Center, Peshawar, Pakistan; 3 Dr. Mujeeb Ur Rehman, MS. Senior Medical Officer, Department of Cardiothoracic Surgery, Rehman Medical Institute, Peshawar, Pakistan; 4 Dr. Bahauddin Khan, FCPS. Assistant Professor, Department of Cardiothoracic Surgery, Northwest General Hospital and Research Center, Peshawar, Pakistan

**Keywords:** IABP, Frequency, Cardiac Surgery, CABG, Left Ventricular Dysfunction

## Abstract

**Objective:**

To find the incidence of intra-aortic balloon pump (IABP) induction and factors associated with its insertion in coronary artery bypass grafting (CABG).

**Methods:**

This retrospective observational non interventional study was conducted at Cardiac Surgery Department, North West General Hospital and Research Center, Peshawar from December 2018 to March 2020. The total sample size was 360 patients who underwent coronary artery bypass grafting (CABG). The research was piloted in the cardiac operation theatre then cardiac intensive care unit (CICU) of Northwest General Hospital and Research center Hayatabad Peshawar. Data was collected from 360 patients scheduled for CABG. Total numbers of patients in whom IABP was inserted and factors associated with IABP insertions were noted. All the information was collected on a specifically prepared Form. Data was entered and evaluated in statistical package for social sciences form 25.

**Results:**

In this study, a total of 360 patients were observed who underwent coronary artery bypass grafting (CABG). We determined the frequency of IABP induction and the factors related to it. Gender distribution among patients who were assisted with IABP was 43% female and 57% male. IABP induction was done for most of moderately to severely reduced ejection fraction patients. Other factors related to patients who required IABP support were previous myocardial infarction 100%, hypertension 86%, diabetes mellitus 64%, coronary end-arterectomy 21% and smoking 7%. The results were analyzed. We have used the (SPSS) version 25 and Chi-square test for analysis in which the P-value less than 0.00001 is statistically significant.

**Conclusion:**

Incidence of insertion of IABP among CABG population was 3.9% in our hospital. It is an essential support to post CABG patient with left ventricular dysfunction after cardiopulmonary bypass with moderate to severely reduced ejection fraction, Myocardial infarction, hypertension, diabetes mellitus. Smoking and endarterectomy were not significantly related to IABP induction in our study. Multicenter study is still required to find out the other factors governing the IABP insertion.

**Abbreviations**
CABG: Coronary Artery Bypass Grafting Surgery
EF: Ejection Fraction
CAD: Coronary Artery Disease
PCI: Percutaneous Coronary Intervention
ICU: Intensive Care Unit
IABP: Intra-aortic Balloon Pump
CICU: Cardiac Intensive Care Unit
MI: Myocardial Infarction

## INTRODUCTION

Coronary artery disease (CAD) has been considered the important cause of death worldwide. Modern techniques in cardiac surgery and perioperative care have drastically reduced the patient’s complications in cardiac surgery but the frequency of this global disease is still high.^1^

Choices for management of CAD comprise medical therapies, percutaneous coronary intervention (PCI) and coronary artery bypass grafting (CABG). Prognosis of CAD is dependent on interventions, baseline personality and genetics.^2^ With the progress of coronary interventional treatment and the popularity of PCI, patients who are experiencing CABG are aged and might have more common coexisting diseases like diabetes mellitus and chronic renal failure which lead to high risk of cardiac surgery.^3^

For cardiac surgery risk evaluation Euro SCORE has been extensively used, which stratifies cardiac surgical risk into three categories: low-risk (0–2 points); intermediate-risk (3–5 points), and high-risk (more than or equal six points.^4^ These high risk patients with coronary artery disease presenting for CABG mostly have left ventricular (LV) dysfunction.^5^ Most of the research has revealed an encouraging survival advantage in patients undergoing surgical treatment of CAD.^6^,^7^ Nevertheless, severe LV dysfunction (ejection fraction ≤ 35%) is stated to be the predictor of low cardiac output syndrome and mortality in CABG.^7^,^8^

Regardless of recent development in treatment, the management of individuals presenting for cardiac surgery with severe left ventricular dysfunction remains a challenge so that mechanical assist devices have been developed to be used in such patients.^9^ One such mechanical assist devices is the intra-aortic balloon pump (IABP) passed via femoral artery that was presented for the first time in 1962 which provides circulatory support.^10^

The IABP is the most frequently used ventricular mechanical support gadget in recent times in patients with ventricular dysfunction making it an immensely beneficial approach in post CABG patients with moderate to severely reduced ejection fraction.^11^ The inflation of IABP occurs during diastole with aortic valve closure synchronously and the presence of dicrotic notch results in displacing of blood into the peripheral circulation from the thoracic aorta which is followed by fast deflation prior the onset of systole period of the cardiac cycle. So IABP can pick up diastolic perfusion pressure, coronary blood flow and myocardial oxygen delivery. It can also reduce left ventricular after load, ventricular work, oxygen expenditure, and encourage cardiac function revitalization.^12^

Our objective was to find the incidence of intra-aortic balloon pump (IABP) induction and factors associated with its insertion in coronary artery bypass grafting (CABG).

## METHODS

This non interventional retrospective study was conducted and approved by ethical committee of Northwest General Hospital and Research Center, a tertiary care hospital located in Hayatabad, Peshawar with Ref. No.: NWGH/EC/18, August 22, 2020. The research was commenced from December 2018 till March 2020, and informed consent was obtained from all the participants. Overall 360 adult patients of both sexes scheduled for CABG surgical procedure were included in the study.

All the necessary pre-operative regular blood tests along with specific investigation for cardiac surgery were performed. Not only on-pump but also off pump cardiopulmonary bypass was performed according to patient’s requirement. During bypass mild to moderate hypothermia was maintained. We used ante grade cold-blooded cardioplegia through the aortic root or the venous graft in our center.

Triggers for insertion of intra-aortic balloon pump after cardiopulmonary bypass in our setup were continuous hypotension with visible cardiac function worsening and signs of tissue hypo perfusion like cold extremities and lowered urine production in spite of elevated inotropic support and fluid boluses. The significance of this study is to keep an eye on special factors leading to IABP insertion like low ejection fraction, DM, MI, HTN.

Comprehensive statistics which include age, gender, procedure, coexisting disease, previous myocardial infarction, left ventricular function according to left ventricular ejection fraction, diseased coronary arteries, intraoperative findings and parameters of participants were documented through a precisely constructed Form afterwards analyzed by using (SPSS) version 25 and Chi-square test for analysis in which the P-value less than 0.00001 is statistically significant.

## RESULTS

Total number of on pump CABG patients was 323 (90%) and off pump CABG was 29 (8%). Other case distribution is shown in Table-I. The intra-aortic balloon pump insertion was in 14 (3.9%) shown in Table-II. IABP was inserted in 6 (43%) female and 8 (57%) male patients. The highest incidence of IABP insertion was significant in patients with moderately reduced left ventricular EF as the P-value is 0.00000 and we have found the same frequency in the severely reduced EF patients. Patients having normal EF and having no need of IABP insertion were compared to moderate and severely reduced EF patients and the results are shown in (Table-III). All the patients who required IABP had previous history of myocardial infarction and it is a very significant factor requiring use of IABP. The use of IABP was more in hypertensive and diabetic patients such as 86% and 64% respectively. Frequency of IABP in Coronary artery endarterectomy is also not significant as P-value is 0.000395. The frequency was much less in smokers which is not significant as P-value is 0.022698 and it was almost same in diabetics, MI and Hypertensive patients as shown in Table-IV.

**Table-I:** Number and percentage of CABG procedures.

*Procedure*	*Number of cases*	*Percentages*
Total number of CABG	360	100%
On Pump CABG	323	90%
Off Pump CABG	29	8%
Redo CABG	2	0.5%
CABG plus Aortic valve replacement	4	1%
CABG plus mitral valve replacement	2	0.5%

**Table-II:** Incidence of insertion of intra-aortic balloon pump (IABP).

*IABP*	*Number*	*Percentage*
Total	360	100%
Inserted	14	3.9%
Not inserted	346	96.1 %

**Table-III:** Frequency of IABP insertion in terms of Ejection Fraction (EF).

*Ejection fraction (EF) %*	*IABP inserted*		

	*Number*	*Percentage*	*P value*
Normal EF>50%	4	28.6%	0.00000
Moderately reduced EF 35-50%	6	42.8%	
Severely reduced EF<35%	4	28.6%	
Total	14	100%	

**Table-IV:** Frequency of IABP insertion on the basis of other most common factors.

*Factors*	*Total number*	*Percentage*	*P value*
Diabetes mellitus	9	64%	0.00000
Hypertension	12	86%	0.00000
Smoker	1	7%	0.022698
Coronary endarterectomy	3	21%	0.000395
Previous myocardial infarction (MI)	14	100%	0.00000

## DISCUSSION

Although surgical intervention is successful in patients with severe coronary artery disease yet, increasing benefits coupled with elevated threat, particularly for older patients with comorbidities like hypertension, diabetes mellitus, chronic obstructive pulmonary disease etc. remain a cause for concern. As a consequence, every contributory factor should be considered and wide-ranging scientific management should be practiced, including improving all system monitoring, cardio respiratory support and IABP incorporation.^13^

The foremost outcomes of IABP insertion are reduction of ventricular after load, enhancement coronary perfusion during diastole and augmentation of subendocardial blood flow resulting in elevated cardiac output and upgraded circulatory outcome. For these reasons, it is highly useful for patients with CABG^14^ regardless of the documented issues associated with IABP such as hemorrhage, vascular damage and platelet dysfunction. It is extensively used in modern era. So as to reduce intra hospital death rate which is as high as 26-50% among cardiac patients, use of IABP support is recommended.^15^ Use of IABP as an urgent measure for postoperative heart failure has time and again showed a survival rate of approximately 70% by many researchers. Another study has established an overall survival of 85% at four years for a cohort of patients who needed IABP supports post-operatively.^16^ Enhanced prognosis after cardiac surgery for coronary revascularization is dependent on preservation of cardiac system subsequent to cardiac operation. IABP has proved to be excellent treatment option for maintenance healthier cardiovascular system after cardiopulmonary bypass.^17^

One of the conditions after coronary artery bypass is stunned myocardium which is brief left ventricular dysfunction due to ischemic event to the heart muscle; IABP has an exceptional benefit in the recovery of this condition.^18^ In literature frequency of IABP application is mentioned 7 %^16^ and in some other studies it is 17% and 22%.^19^ It was reported 11.1% in the recently conducted local study.^20^

The use of IABP insertion of 3.9 % in our study is far less than the figures reported in literature and we expect that it may be just because of small study. Average age of patients who were subjected to IABP insertion in our study was 59.8 years with SD ± 1.26 as compared to scientific literature, use of IABP is generally applied in older age CABG patients and it is also similar to the local research 58.01 ±9.126.^20^,^21^

Incidence of hypertension was 86% in our research which is almost similar to reported studies.^22^ Another local study has also reported the higher number of hypertensive patients in IABP group.^20^ The other coexisting disease in this cohort of patients was diabetes that was 64% which is also very high and is reported 41.1% in other study.^23^ Elevated incidence of IABP use in diabetics might be due to the fact that these patients not only have larger scale of preexisting atherosclerosis but also elevated frequency of restenosis which leads to a higher percentage of myocardium unguarded and poorly re-vascularized so a larger percentage of the myocardium becomes ischemic.^24^

Incidence of previous myocardial infarction in our study was 100% in participants requiring IABP. This is comparable to the reported incidence in another study.^25^ It is still controversial as in another study recently it was reported 14.8%. We noticed in our research enhanced hemodynamic stability after initiation of IABP in patients with previous myocardial infarction making it very beneficial to use IABP in such patients.

We noticed in our research that incidence of IABP use was more in patient with moderately impaired left ventricle and it is reported 39.9% in other study. We reported the same frequency in both normal and severely reduced left ventricular fraction patients which is contrary to other studies which show less incidence of IABP in normal EF patients. These findings are somewhat controversial to those reported by Shah SMA, et al.^20^ showing higher incidence of IABP use in moderately reduced or severely reduced ejection fraction and was reduced in normal or good left ventricular function.

Our study further showed that incidence of IABP application does not increase with decreasing ejection fraction rather a good percentage of patients with normal ejection fraction required IABP which is contrary to what is reported.^26^ This is due to the fact that ejection fractions mentioned in echocardiography sometimes does not match with the actual ejection fraction of patients. We reported the mortality rate 35.7% which is comparable with those reported in the literature.^27^ Local study by Shah SMA, et al.^20^ has reported the mortality rate of 4.3%.

This higher mortality rate does not indicate that it was related IABP but it was due to critical condition and coexisting diseases in this group of patients. In other words IABP benefited 64% of critically ill patients. Mortality rate was 1.44% in patients without IABP. However, ICU stay and ventilator time was prolonged in IABP group which is similar to the findings of He XY.^25^

The scientific importance of our research is that application of IABP at any time during surgical intervention in patients with coronary artery disease having left ventricular dysfunction produces improved results due to on time support to myocardium and other vital organs prior to irreparable injury.

### Limitation of the study

This study is based on small sample and it was performed to know only the incidence of IABP insertion in CABG patients during and after weaning from cardiopulmonary bypass and reported with few factors associated with IAPB use. Another study with larger sample size can be conducted with more variables among different groups for comparison and statistical significance.

## CONCLUSION

IABP was mostly used in moderately reduced EF patients but also used in patients with good ventricular function when needed in CABG surgery with considerably excellent results. It is lifesaving mechanical support. In our study, IABP use was also considered in patients with recent MI, hypertensive and diabetic patients.

### Authors’ Contribution:

**IA and MUR:** Conceived, designed, did statistical analysis. manuscript writing and are responsible for integrity of the study.

**MUI:** Did data collection and editing of manuscript.

**BK:** Did review and final approval of manuscript.
